# Association between socioeconomic position and cystatin C in the Heinz Nixdorf Recall Study

**DOI:** 10.1038/s41598-021-98835-7

**Published:** 2021-09-29

**Authors:** Tanja Zamrik, Mirjam Frank, Carina Emmel, Lars Christian Rump, Raimund Erbel, Karl-Heinz Jöckel, Nico Dragano, Börge Schmidt

**Affiliations:** 1 Institute for Medical Informatics, Biometry and Epidemiology, University Hospital Essen, University of Duisburg-Essen, Hufelandstr. 55, 45122 Essen, Germany; 2 Clinic of Nephrology, University Hospital Düsseldorf, Düsseldorf, Germany; 3 Institute of Medical Sociology, Centre for Health and Society, University Hospital Düsseldorf, Düsseldorf, Germany

**Keywords:** Biomarkers, Risk factors, Epidemiology

## Abstract

Social inequalities in health and disease are well studied. Less information is available on inequalities in biomarker levels indicating subclinical stages of disease such as cystatin C, an early diagnostic marker of renal dysfunction and predictor for cardiovascular disease. We evaluated the relationship between cystatin C, socioeconomic position (SEP) and established cardiovascular risk factors in a population-based study. In 4475 men and women aged 45–75 years participating in the baseline examination of the Heinz Nixdorf Recall Study cystatin C was measured from serum samples with a nephelometric assay. SEP was assessed by education and household income. Linear regression models were used to analyse the association between SEP and cystatin C as well as the impact of cardiovascular risk factors (i.e., body mass index, blood pressure, blood glucose, diabetes mellitus, blood lipids, C-reactive protein, smoking) on this association. After adjustment for age and sex cystatin C decreased by 0.019 mg/l (95% confidence interval (CI) − 0.030 to − 0.008) per five years of education. While using a categorical education variable cystatin C presented 0.039 mg/l (95% CI 0.017–0.061) higher in men and women in the lowest educational category (≤ 10 years of education) compared to the highest category (≥ 18 years). Concerning income, cystatin C decreased by 0.014 mg/l (95% CI − 0.021 to − 0.006) per 1000 € after adjustment for age and sex. For men and women in the lowest income quartile cystatin C was 0.024 mg/l (95% CI 0.009–0.038) higher compared to the highest income quartile. After adjusting for established cardiovascular risk factors the observed associations were substantially diminished. Social inequalities seem to play a role in subclinical stages of renal dysfunction, which are also related to development of cardiovascular disease. Adjustment for traditional cardiovascular risk factors showed that these risk factors largely explain the association between SEP and cystatin C.

## Introduction

According to World Health Organization cardiovascular diseases (CVD) globally represent the main cause of death^1^. Therefore, prevention and early recognition is highly relevant. Previous research findings have shown an association of cardiovascular risk with socioeconomic factors. Men and women of lower socioeconomic position (SEP) do not only suffer more frequently from coronary artery disease^2^, but also from higher levels of coronary artery calcification as a marker of subclinical atherosclerosis^3^. Diverse approaches exist in order to explain this relationship; material, behavioural and psychosocial factors have to be considered^4^. For gaining knowledge on how socioeconomic factors influence biological pathways of cardiovascular risk, it is important to quantify the impact of SEP on biomarkers indicating subclinical stages of cardiovascular outcomes. In this context, determining the role of known and modifiable cardiovascular risk factors may help to take early preventive and therapeutic actions.

There is evidence from epidemiological studies for an association between increased cystatin C levels, as diagnostic marker of subclinical renal dysfunction, and cardiovascular mortality^5^, which has been described as being stronger compared to the association with creatinine^6^. However, after adjusting for traditional cardiovascular risk factors and hs-CRP, an independent association between cystatin C and cardiovascular disease has been observed in most but not all studies^7–9^. Additionally, Mendelian randomization analyses did not support a causal role of cystatin C in the ethology of CVD^9,10^. The relationship between cystatin C and CVD could likely be explained (1) by cystatin C being a marker for renal dysfunction, which itself contributes to increased risk of cardiovascular disease or (2) by its correlation with long-term exposure to CVD risk factors such as hypertension or diabetes^10^. In a cohort study by Kestenbaum et al. cystatin C has been associated with a greater incidence of hypertension^11^. In Shankar and Teppala this association has been found for women, but not for men in a cross-sectional study design^12^. A systematic review and meta-analysis by Ma et al. demonstrated that type 2 diabetes mellitus patients had higher cystatin C compared to healthy controls^13^. In a meta-analysis by van der Laan et al. cystatin C was associated with several cardiovascular risk factors and traits like LDL, HDL, BMI, diastolic blood pressure and smoking. In their genetic analysis however, the cystatin C associated SNP (rs911119) showed no significant association with these traits^9^. A mendelian randomization study by Rasheed et al. did not observe a causal effect of kidney function measured by estimated glomerular filtration rate (GFR) on lipids^14^.

On the other hand, increased levels of cystatin C seem to be associated with systemic inflammation which may indicate an increase in mortality risk independently of GFR^15^. Some authors have described the prognostic benefit of cystatin C as marker of coronary artery disease severity and long-term mortality (both cardiovascular and non-cardiovascular) in populations with normal renal function^16,17^, even though therapeutics targeted at lowering circulating cystatin C are unlikely to be effective in preventing CVD^9^.

It is assumed, that the effect of SEP on cardiovascular morbidity and mortality is not direct, but mediated through a complex interplay of social inequalities in risk factors^18^. Evidence from longitudinal studies had shown that the CVD risk factors smoking^19,20^, and systolic blood pressure^21,22^, are associated with cystatin C, both in the presence and absence of kidney disease. An association of BMI and cystatin C could not be seen in longitudinal^23^ but in cross-sectional study design^24^. A mendelian randomization study has given some evidence for a weak causal effect of triglycerides, HDL but not LDL on estimated GFR using cystatin C^14^. As people with low SEP tend to have a worse CVD risk profile including higher prevalence of smoking and high blood pressure, the role of these risk factors as mediators in a possible relationship between SEP and cystatin C as a marker of renal disease and predictor for CVD is of interest.

While there are some studies describing the relationship between indicators of SEP and chronic kidney disease^25^, information on the association between SEP and cystatin C considering the impact of established cardiovascular risk factors is sparse. The aim of the present study was to investigate the impact of educational attainment and household income on levels of cystatin C in a population-based cohort study separately for women and men, while including cardiovascular risk factors as potential mediators in the analysis.

## Methods

### Study population

Baseline data from the Heinz Nixdorf Recall Study were used, including 4814 men and women aged 45–75 years recruited in three large cities in the western part of Germany (Bochum, Essen and Mülheim/Ruhr) during 2000–2003^26,27^. The participants were selected by random sampling from registration offices with a baseline response rate of 56%^28^. In the course of the study medical histories, clinical examinations, cardiological functional diagnostics, imaging as well as information about cardiovascular risk factors, psychosocial and socioeconomic factors were gathered. Furthermore, urine and blood samples were collected and subsequently frozen in a biobank to enable future examinations. All participants provided written informed consent and the study was approved by the ethical committee of the University of Duisburg-Essen. The Heinz Nixdorf Recall Study was certified and recertified by DIN ISO 9001:2000/2008 and is performed in accordance with the Declaration of Helsinki.

### Cystatin C

Cystatin C at study baseline was measured in 2012 from frozen blood samples at the laboratory of the University Hospital in Düsseldorf (Germany). Cystatin C was quantified by particle-enhanced immunonephelometric assay (Siemens, Marburg, Germany). The manufacturer indicated the analytical sensitivity of the assay by 0.05 mg/l^29^.

### Socioeconomic position

Education and income were used as SEP indicators assessed at study baseline by standardized face-to-face interviews. Education was defined according to the International Standard Classification of Education (ISCED) as total number of educational years consisting of highest school and professional degree^30^. In order to categorize the educational variable four groups were created with the lowest category including 10 or less years of educational training and the highest 18 or more years.

Income was calculated as monthly household income in Euros by dividing the total net income of a household by a weighting factor for each household member^31^. For a categorized presentation income was divided into four groups based on the calculated quartiles of the analyzed population.

### Cardiovascular risk factors

Established cardiovascular risk factors (i.e. body mass index (BMI), blood pressure, diabetes, smoking, total cholesterol, high-density lipoprotein (HDL), low-density lipoprotein (LDL), triglycerides and high-sensitive C-reactive protein (hs-CRP)) assessed at study baseline were included in the analyses. BMI was determined based on standardized direct measurement of weight and height (kg/m^2^). Blood pressure was measured using an oscillometric device (Omron, HEM-705CP, OMRON Corporation, Hoofdrop, the Netherlands) and the mean of the second and third of three measurements was calculated. Blood pressure was classified in accordance with the guidelines of JNC7 (The Seventh Report of the Joint National Committee on Prevention, Detection, Evaluation, and Treatment of High Blood Pressure)^32^. Hypertension was defined as high blood pressure stage 1 and 2 according to JNC7 or the intake of antihypertensive medication. Diabetes was considered as self-reported diagnosis, the intake of antidiabetic drugs or an elevation of fasting or non-fasting glucose. Blood glucose was determined by enzymatic hexokinase method. Smoking was assessed in face-to-face interviews and divided in three categories including ‘current smoker’, ‘ex-smoker’ and ‘never smoked’. Total cholesterol, HDL, LDL and triglycerides were assessed from serum samples by standardized enzymatic methods. Hs-CRP was measured from serum samples with the BN-II nephelometer (Dade Behring/ Siemens Healthcare Diagnostics, Eschborn, Germany).

### Statistical analyses

Out of the 4814 participants of the Heinz Nixdorf Recall Study serum cystatin C levels were missing for 339. Furthermore, we excluded three participants with cystatin C levels > 4 mg/l in order to avoid outliers. Some observations on education (n = 12) and income (n = 282) were missing, but no correlation between missing cystatin C and SEP was found. There were also missing data for some of the regarded risk factors (see Table 1).

**Table 1:** Characteristics of the analysis population (M: mean; SD: standard deviation; IQR: interquartile range; [n_miss_ = number of missings]).

	All	Men	Women
N (%)	4472 (100%)	2224 (49.7%)	2248 (50.3%)
**Age (years)**			
M ± SD [n_miss_ = 0]	59.6 ± 7.8	59.6 ± 7.8	59.5 ± 7.8
**Cystatin C (mg/l)**			
M ± SD	0.78 ± 0.18	0.81 ± 0.19	0.75 ± 0.17
**Education (years of training) N (%) [n**_**miss**_** = 12]**			
≤ 10	504 (11.3%)	112 (5.1%)	392 (17.5%)
11–13	2490 (55.8%)	1063 (48.0%)	1427 (63.6%)
14–17	994 (22.3%)	741 (33.5%)	253 (11.3%)
≥ 18	472 (10.6%)	299 (13.5%)	173 (7.7%)
**Income (Euro/month)**			
Median (IQR) [n_miss_ = 282]	1449 (1108–1875)	1520 (1108–2073)	1313 (937–1875)
**Coronary artery disease at baseline**			
N (%) [n_miss_ = 11]	298 (6.7%)	235 (10.6%)	63 (2.8%)
**Stroke at baseline**			
N (%) [n_miss_ = 25]	123 (2.8%)	76 (3.4%)	47 (2.1%)
**BMI (kg/m**^**2**^**)**			
M ± SD [n_miss_ = 23]	27.9 ± 4.7	28.2 ± 4.0	27.7 ± 5.2
**Systolic blood pressure (mmHg)**			
M ± SD [n_miss_ = 11]	133.1 ± 20.7	138.1 ± 19.3	128.2 ± 21.0
**Diastolic blood pressure (mmHg)**			
M ± SD [n_miss_ = 12]	81.4 ± 10.9	83.9 ± 10.5	79.0 ± 10.6
**Hypertension**			
N (%) [n_miss_ = 12]	2543 (57.0%)	1398 (63.1%)	1145 (51.0%)
**Glucose (mg/dl)**			
M ± SD [n_miss_ = 3]	111.8 ± 28.8	115.9 ± 32.1	107.6 ± 24.6
**Diabetes mellitus**			
N (%) [n_miss_ = 0]	602 (13.5%)	380 (17.1%)	22 (9.9%)
**Hs-CRP (mg/dl)**			
Median (IQR) [n_miss_ = 9]	0.15 (0.07–0.33)	0.15 (0.07–0.32)	0.15 (0.07–0.33)
**Total cholesterol (mg/dl)**			
M ± SD [n_miss_ = 1]	229.1 ± 39.1	225.0 ± 38.3	233.1 ± 39.5
**HDL (mg/dl)**			
M ± SD [n_miss_ = 2]	58.1 ± 17.2	51.1 ± 14.3	65.0 ± 16.9
**LDL (mg/dl)**			
M ± SD [n_miss_ = 15]	145.5 ± 36.2	145.3 ± 35.7	145.7 ± 36.8
**Triglycerides (mg/dl)**			
M ± SD [n_miss_ = 4]	149.1 ± 102.2	165.6 ± 119.6	132.8 ± 78.1
**Active smokers**			
N (%) [n_miss_ = 6]	1030 (23.1%)	553 (24.9%)	447 (21.2%)

Describing the data, for quantitative variables mean ± standard deviation or median and interquartile range (IQR) were stated. Qualitative variables were indicated by absolute (N) and relative frequencies (%). Multiple linear regression was applied to quantify the association between SEP, cardiovascular risk factors and cystatin C. Income and education were included separately as continuous as well as categorical independent variables in the regression models. The highest SEP category served as a reference in the categorical analysis. Different regression models were calculated including a crude model (unadjusted) and a basic model (adjusted for the confounders age and sex) to estimate the total effect of SEP on cystatin C. Using education and income as continuous variables, the basic model was separately supplemented by each cardiovascular risk factor hypothesized as potential mediators for the effect of SEP on cystatin C. Finally, a full model adjusted for BMI, hypertension, diabetes, hs-CRP, total cholesterol, HDL, triglycerides and smoking was calculated to estimate the direct effect of SEP on cystatin C.

Sensitivity analyses were performed by excluding participants with coronary artery disease, stroke or GFR < 60 ml/min/1.73 m^2^ at baseline from the study population in order to examine the robustness of the results in a population without manifest cardiovascular diseases or impaired renal function.

All analyses were stratified by sex. All calculations were computed using the IBM software SPSS Statistics Version 25 (SPSS Inc., Chicago, Illinois, USA).

## Results

In men, cystatin C showed higher levels compared to women (Table 1). Men also held a higher educational level as well as higher income. Most cardiovascular risk factors were more pronounced in men compared to women at the same average age.

**Figure 1:**
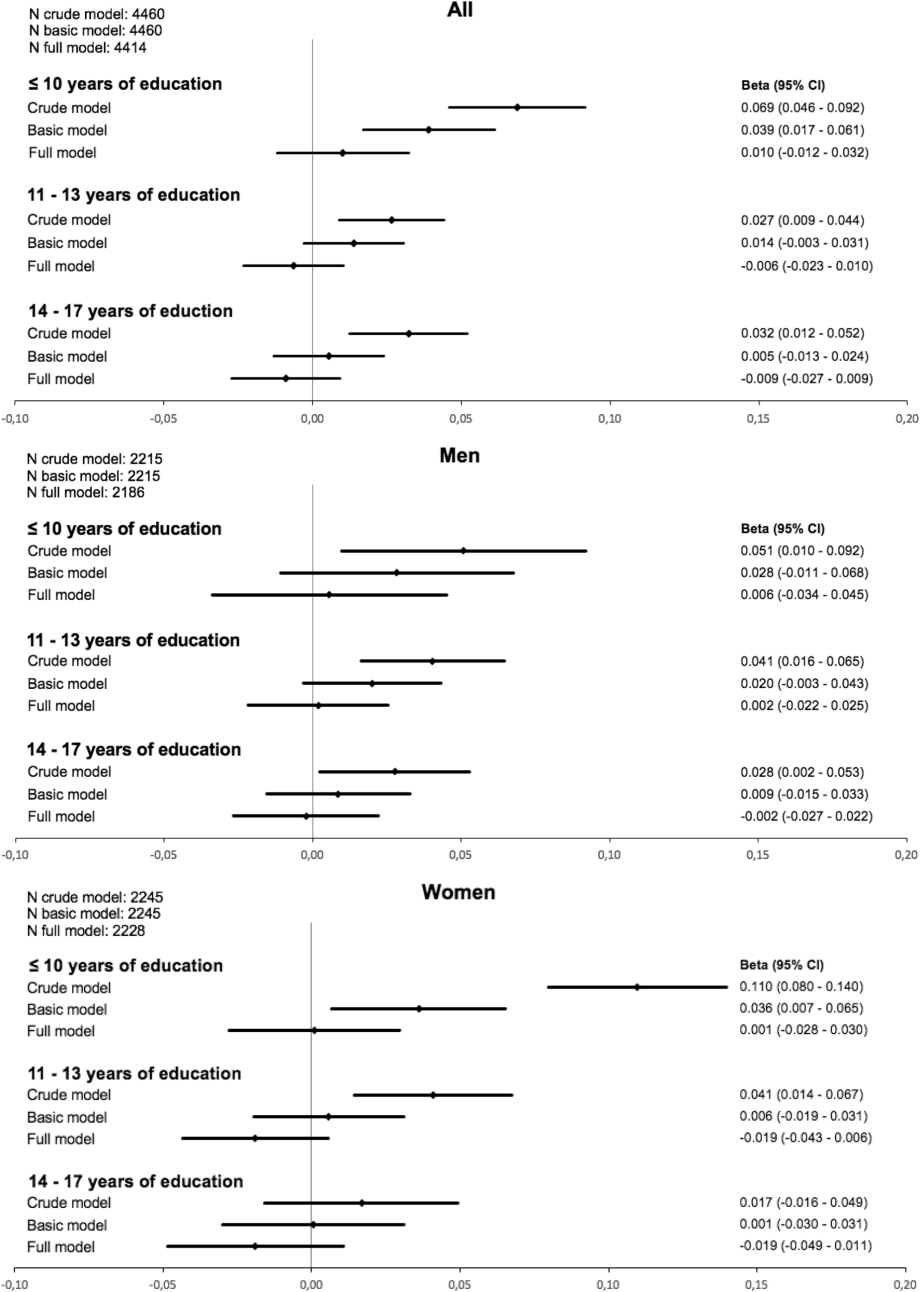
Beta estimates and 95% confidence intervals (95% CI) for the association of education categories (≥ 18 years of education as reference) with cystatin C in linear regression models (basic model: adjusted for age (+ sex); full model: age (+ sex), BMI, hypertension, diabetes, hs-CRP, total cholesterol, HDL, triglycerides, smoking).

Considering the lowest category of education, in the overall group a 0.039 mg/l higher level of cystatin C was observed compared to the highest category after adjusting for age and sex (Fig. 1). Across all subgroups the effect estimates were strongest for the lowest compared to the highest educational category and decreased almost gradually with increasing categories. There was a successive decline in effects from the crude model over the basic model to the full model. After adjustment for cardiovascular risk factors the effect of education on cystatin C was substantially reduced in both men and women. The difference in effect estimates between the crude and the basic model in the female subgroup was strongest, especially in the lowest educational category.

**Figure 2:**
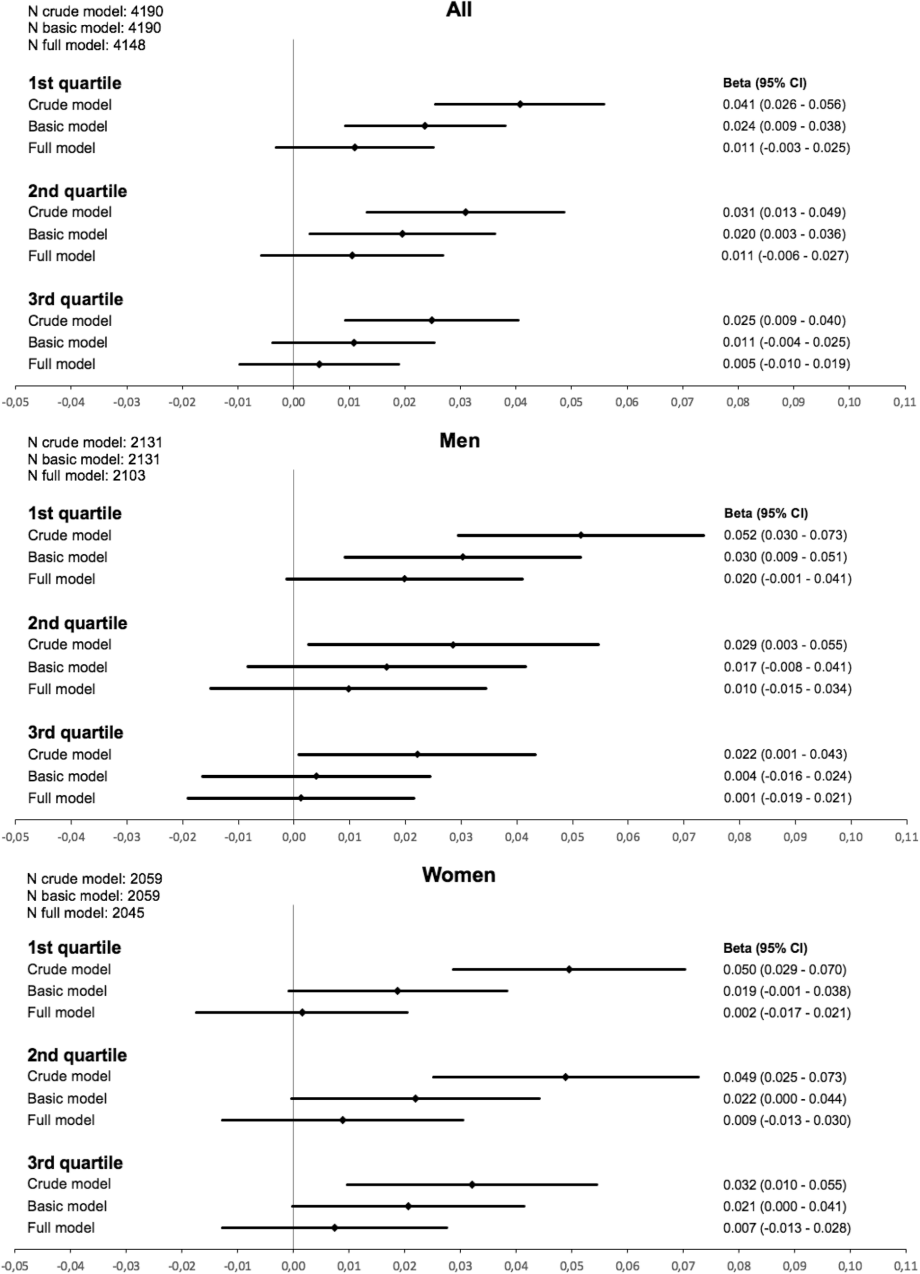
Beta estimates and 95% confidence intervals (95% CI) for the association of income quartiles (4th quartile as reference) with cystatin C in linear regression models (basic model: adjusted for age (+ sex); full model: age (+ sex), BMI, hypertension, diabetes, hs-CRP, total cholesterol, HDL, triglycerides, smoking).

Concerning income as SEP indicator, similar observations were made (Fig. 2). For the overall group a 0.024 mg/l higher level of cystatin C was observed for the lowest income quartile compared to the highest after adjusting for age and sex. The effect estimates in the lowest quartile presented strongest. Using the full model, the effect of income on cystatin C was substantially reduced.

For the crude linear regression model using the continuous education variable as SEP indicator, effect estimates were considerably higher in women than in men (Table 2). Here, cystatin C was on average 0.064 mg/l lower per five educational years. After adjustment for age (and sex in the overall group) the effect estimates diminished with the strongest decrease in women. Adjustment for single risk factors displayed different results. After adjustment for BMI, hypertension, HDL, triglycerides and smoking a reduction of effect strength was observed with BMI showing the highest impact. In the full model the strength of association between education and cystatin C was further reduced.

**Table 2:** Beta estimates and 95% confidence intervals (95% CI) for the association of education (per 5 years of education) with cystatin C in linear regression models (basic model: adjusted for age (+ sex); full model: age (+ sex), BMI, hypertension, diabetes, hs-CRP, total cholesterol, HDL, triglycerides, smoking).

	All			Men			Women		
	N	β	95% CI	N	β	95% CI	N	β	95% CI
Crude model	4460	− 0.023	− 0.035 to − 0.012	2215	− 0.026	− 0.043 to − 0.009	2245	− 0.064	− 0.080 to − 0.048
Basic model	4460	− 0.019	− 0.030 to − 0.008	2215	− 0.017	− 0.033 to 0.000	2245	− 0.020	− 0.036 to − 0005
Basic + BMI	4439	− 0.011	− 0.022 to 0.000	2205	− 0.012	− 0.029 to 0.004	2234	− 0.008	− 0.023 to 0.007
Basic + systolic blood pressure	4454	− 0.019	− 0.030 to − 0.007	2211	− 0.016	− 0.033 to 0.000	2243	− 0.020	− 0.036 to − 0.005
Basic + diastolic blood pressure	4455	− 0.019	− 0.030 to − 0.008	2211	− 0.016	− 0.033 to 0.000	2244	− 0.021	− 0.036 to − 0.005
Basic + hypertension	4449	− 0.017	− 0.028 to − 0.005	2207	− 0.014	− 0.030 to 0.002	2242	− 0.018	− 0.034 to − 0.003
Basic + glucose	4457	− 0.019	− 0.030 to − 0.007	2213	− 0.016	− 0.033 to 0.000	2244	− 0.020	− 0.035 to − 0.004
Basic + diabetes	4460	− 0.018	− 0.029 to − 0.007	2215	− 0.016	− 0.032 to 0.000	2245	− 0.018	− 0.034 to − 0,003
Basic + hs-CRP	4451	− 0.018	− 0.029 to − 0.007	2208	− 0.015	− 0.031 to 0.001	2243	− 0.020	− 0.035 to − 0.004
Basic + total cholesterol	4459	− 0.019	− 0.030 to − 0,008	2214	− 0.016	− 0.033 to 0.000	2245	− 0.020	− 0.036 to − 0.005
Basic + HDL	4458	− 0.014	− 0.025 to − 0.003	2213	− 0.014	− 0.031 to 0.002	2245	− 0.013	− 0.028 to 0.002
Basic + LDL	4445	− 0.019	− 0.030 to − 0.007	2207	− 0.017	− 0.033 to 0.000	2238	− 0.020	− 0.036 to − 0.005
Basic + triglycerides	4456	− 0.017	− 0.028 to − 0.006	2212	− 0.016	− 0.032 to 0.001	2244	− 0.017	− 0.032 to − 0,001
Basic + smoking	4460	− 0.016	− 0.027 to − 0.004	2215	− 0.011	− 0.028 to 0.005	2245	− 0.019	− 0.034 to − 0.003
Full model	4414	− 0.005	− 0.016 to 0.006	2186	− 0.004	− 0.020 to 0.013	2228	− 0.003	− 0.019 to 0.012

Analogous results for income (continuous) are described in Table 3. Again, the crude model showed stronger effect estimates in women than in men, which were reduced strongest in the highest income quartile by adjustment for age. In the overall group, cystatin C was on average 0.014 mg/l lower per 1000 euros income in the basic model. After adjusting for single risk factors in the overall group, for BMI, hypertension, diabetes, hs-CRP, HDL and smoking a reduction of effect strength was observed. Adjustment for all considered risk factors caused further reduction of effects.

**﻿Table 3:** Beta estimates and 95% confidence intervals (95% CI) for the association of income (per 1000 Euro/month) with cystatin C in linear regression models (basic model: adjusted for age (+ sex); full model: age (+ sex), BMI, hypertension, diabetes, hs-CRP, total cholesterol, HDL, triglycerides, smoking).

	All			Men			Women		
	N	β	95% CI	N	β	95% CI	N	β	95% CI
Crude model	4190	− 0.020	− 0.028 to − 0.012	2131	− 0.022	− 0.034 to − 0.011	2059	− 0.028	− 0.039 to − 0.017
Basic model	4190	− 0.014	− 0.021 to − 0.006	2131	− 0.015	− 0.026 to − 0.004	2059	− 0.012	− 0.022 to − 0.002
Basic + BMI	4171	− 0.010	− 0.017 to − 0.003	2121	− 0.013	− 0.024 to − 0.002	2050	− 0.006	− 0.016 to 0.004
Basic + systolic blood pressure	4185	− 0.014	− 0.022 to − 0.007	2127	− 0.015	− 0.026 to − 0.005	2058	− 0.012	− 0.023 to − 0.002
Basic + diastolic blood pressure	4186	− 0.014	− 0.021 to − 0.007	2127	− 0.015	− 0.026 to − 0.004	2059	− 0.012	− 0.023 to − 0.002
Basic + hypertension	4180	− 0.012	− 0.020 to − 0.005	2123	− 0.014	− 0.024 to − 0.003	2057	− 0.010	− 0.021 to 0.000
Basic + glucose	4187	− 0.014	− 0.021 to − 0.006	2129	− 0.015	− 0.026 to − 0.004	2058	− 0.012	− 0.022 to − 0.002
Basic + diabetes	4190	− 0.013	− 0.021 to − 0.006	2131	− 0.015	− 0.026 to − 0.004	2059	− 0.011	− 0.021 to − 0.001
Basic + hs-CRP	4182	− 0.013	− 0.021 to − 0.006	2125	− 0.014	− 0.025 to − 0.003	2057	− 0.012	− 0.022 to − 0.002
Basic + total cholesterol	4189	− 0.014	− 0.021 to − 0.006	2130	− 0.015	− 0.026 to − 0.004	2059	− 0.012	− 0.023 to − 0.002
Basic + HDL	4188	− 0.012	− 0.019 to − 0.004	2129	− 0.014	− 0.025 to − 0.003	2059	− 0.008	− 0.019 to 0.002
Basic + LDL	4176	− 0.014	− 0.021 to − 0.006	2123	− 0.015	− 0.025 to − 0.004	2053	− 0.012	− 0.022 to − 0.002
Basic + triglycerides	4186	− 0.014	− 0.021 to − 0.006	2128	− 0.015	− 0.026 to − 0.004	2058	− 0.011	− 0.021 to − 0.001
Basic + smoking	4190	− 0.012	− 0.020 to − 0.005	2131	− 0.013	− 0.024 to − 0.002	2059	− 0.011	− 0.021 to − 0.001
Full model	4148	− 0.007	− 0.014 to 0.0002	2103	− 0.010	− 0.020 to 0.001	2045	− 0.003	− 0.013 to 0.007

Tables S1–S4 in the supplement show the results of sensitivity analysis which refers to those participants without coronary artery disease or stroke and a GFR ≥ 60 ml/min/1.73 m^2^ at baseline. The trends observed in the main analysis population can also be found in the sensitivity analysis, although effect estimates were less strong.

## Discussion

The results of the present study demonstrated an association between SEP indicators education and income with cystatin C. Participants with lower SEP presented higher cystatin C levels after adjustment for confounding by age and sex. After adjusting for traditional cardiovascular risk factors, this association was strongly attenuated suggesting that known social inequalities in cardiovascular risks mediate the association between SEP and cystatin C. Accordingly, people with lower SEP more frequently hold adverse risk factor profiles which also seemed to be associated with higher levels of cystatin C. Substantial sex-differences in the association between SEP and cystatin C were not observed. Sensitivity analysis demonstrated that the results were not strongly influenced by participants with manifest cardiovascular diseases or impaired renal function, demonstrating that the observed association between SEP indicators education and income with cystatin C is not mainly triggered by social inequalities in Cystatin C-related clinical endpoints, but is also indicated at the subclinical stage.

Previous studies have produced convincing evidence for an inverse relationship between SEP and manifest kidney disease^33^, but few studies have examined the association between SEP and cystatin C as a subclinical biomarker for early kidney disease. Peralta et al. (2006) have reported an association between income and cystatin C in African Americans consistent to the results of the present study^34^. Tamrat et al. have used cystatin C based estimated GFR for their study in African Americans and reported an association with income corresponding to the results of the present study^35^. With regard to European study populations, Thio et al. have published results from the Netherlands showing a longitudinal association between education and impaired renal function (estimated GFR including serum creatinine and cystatin C)^36^. Consistent to the results presented here, the association of education and renal dysfunction has been largely explained by mediation through traditional cardiovascular risk factors as diabetes, overweight, smoking and hypertension^36^. In a population-based cross-sectional study in Ireland Canney et al. (2018) have found a strong association between low childhood SEP and chronic kidney disease defined by a GFR < 60 ml/min/1.73 m^2^ in women including cystatin C for GFR estimation. In contrast to the study presented here, an association between SEP and CKD in men was not observed^37^. Adding the results presented here to previous evidence, early recognition of high levels of cystatin C combined with reduction of modifiable cardiovascular risk factors seems to offer great potential to lower the impact of socioeconomic inequalities on Cystatin C as a marker for renal dysfunction on the subclinical level.

While having the strength of a population-based study design with available information on different SEP indicators and a wide range of cardiovascular risk factors, this study also has some limitations. First, the cross-sectional design has to be considered. This prohibits strong conclusions about the causal direction of the association between income and cystatin C. However, concerning education reversed causation is very unlikely, because it represents a stable indicator for SEP across the life course, usually determined in young adulthood. Using subclinical measures of health further reduces the potential extent of reverse causation. However, reverse causation in the relationship of cystatin C and blood pressure is possible, as there is some evidence from previous studies that cystatin C may be caused by hypertension and vice versa. Therefore, the cross-sectional design of the present study does not allow to draw conclusion about the potential role of hypertension mediating the association between SEP and cystatin C. Results of the sensitivity analysis showed that prevalent cases of cardiovascular disease or renal impairment did not strongly affect the observed associations. Another limitation was that at the time of laboratory analysis standardized assays for cystatin C blood level determination were not yet available. This may limit the comparability of absolute cystatin C values with other studies.

Despite these limitations the results of the present study demonstrate the impact of socioeconomic factors on cystatin C and show that the association is also apparent when cystatin C is used as a subclinical marker in participants without clinical endpoints such as renal impairment, coronary artery disease and stroke. Consideration of traditional cardiovascular risk factors suggests that prevention strategies to modify adverse health behaviors could also be helpful to reduce levels of cystatin C, especially in groups of lower SEP.

## Supplementary Information

Supplementary Tables.

## Data Availability

Due to data security reasons (i.e., data contain potentially participant identifying information), the Heinz Nixdorf Recall Study does not allow sharing data as a public use file. However, others can access the data used upon request, which is the same way authors of the present paper obtained the data. Data requests can be addressed to: recall@uk-essen.de.

## References

[1] WHO. *The top 10 causes of death*. http://www.who.int/mediacentre/factsheets/fs310/en/. (2017).

[2] Bashinskaya B, Nahed BV, Walcott BP, Coumans JV, Onuma OK (2012). Socioeconomic status correlates with the prevalence of advanced coronary artery disease in the United States. PLoS ONE.

[3] Dragano N (2007). Subclinical coronary atherosclerosis is more pronounced in men and women with lower socio-economic status: Associations in a population-based study. Coronary atherosclerosis and social status. Eur. J. Cardiovasc. Prev. Rehabil..

[4] Moor I, Spallek J, Richter M (2017). Explaining socioeconomic inequalities in self-rated health: A systematic review of the relative contribution of material, psychosocial and behavioural factors. J. Epidemiol. Community Health.

[5] Luo J (2015). Cystatin C and cardiovascular or all-cause mortality risk in the general population: A meta-analysis. Clinica Chimica Acta Int J. Clin. Chem..

[6] Astor BC (2009). Method of glomerular filtration rate estimation affects prediction of mortality risk. J. Am. Soc. Nephrol..

[7] Toft I (2011). Cystatin C as risk factor for cardiovascular events and all-cause mortality in the general population. The Tromsø Study. Nephrol. Dialysis Transplant..

[8] Ix JH, Shlipak MG, Chertow GM, Whooley MA (2007). Association of cystatin C with mortality, cardiovascular events, and incident heart failure among persons with coronary heart disease: Data from the Heart and Soul Study. Circulation.

[9] van der Laan SW (2016). Cystatin C and cardiovascular disease: A Mendelian randomization study. J. Am. Coll. Cardiol..

[10] Svensson-Farbom P (2015). Cystatin C is not causally related to coronary artery disease. PLoS ONE.

[11] Kestenbaum B (2008). Differences in kidney function and incident hypertension: The multi-ethnic study of atherosclerosis. Ann. Intern. Med..

[12] Shankar A, Teppala S (2011). Relationship between serum cystatin C and hypertension among US adults without clinically recognized chronic kidney disease. J. Am. Soc. Hypertens..

[13] Ma CC, Duan CC, Huang RC, Tang HQ (2020). Association of circulating cystatin C levels with type 2 diabetes mellitus: A systematic review and meta-analysis. Arch. Med. Sci..

[14] Rasheed H (2021). The causal effects of serum lipids and apolipoproteins on kidney function: Multivariable and bidirectional Mendelian-randomization analyses. Int. J. Epidemiol..

[15] Reese PP, Feldman HI (2009). More evidence that cystatin C predicts mortality better than creatinine. J. Am. Soc. Nephrol..

[16] Wang GN (2014). Serum cystatin C levels are associated with coronary artery disease and its severity. Clin. Biochem..

[17] Wu CK (2010). Cystatin C and long-term mortality among subjects with normal creatinine-based estimated glomerular filtration rates: NHANES III (Third National Health and Nutrition Examination Survey). J. Am. Coll. Cardiol..

[18] Schultz WM (2018). Socioeconomic status and cardiovascular outcomes: Challenges and interventions. Circulation.

[19] Funamoto M (2019). Serum cystatin C, a sensitive marker of renal function and cardiovascular disease, decreases after smoking cessation. Circ. Rep..

[20] Drummond CA (2017). Cigarette smoking and cardio-renal events in patients with atherosclerotic renal artery stenosis. PLoS ONE.

[21] Bloomfield GS (2013). Blood pressure and chronic kidney disease progression in a multi-racial cohort: The Multi-Ethnic Study of Atherosclerosis. J. Hum. Hypertens..

[22] Judson GL (2018). Longitudinal blood pressure changes and kidney function decline in persons without chronic kidney disease: Findings from the MESA study. Am. J. Hypertens..

[23] de Boer IH (2009). Obesity and change in estimated GFR among older adults. Am. J. Kidney Dis..

[24] Stevens LA (2009). Factors other than glomerular filtration rate affect serum cystatin C levels. Kidney Int..

[25] Nicholas SB, Kalantar-Zadeh K, Norris KC (2015). Socioeconomic disparities in chronic kidney disease. Adv. Chronic Kidney Dis..

[26] Erbel R (2010). Coronary risk stratification, discrimination, and reclassification improvement based on quantification of subclinical coronary atherosclerosis: The Heinz Nixdorf Recall study. J. Am. Coll. Cardiol..

[27] Schmermund A (2002). Assessment of clinically silent atherosclerotic disease and established and novel risk factors for predicting myocardial infarction and cardiac death in healthy middle-aged subjects: Rationale and design of the Heinz Nixdorf RECALL Study. Risk factors, evaluation of coronary calcium and lifestyle. Am. Heart J..

[28] Stang A (2005). Baseline recruitment and analyses of nonresponse of the Heinz Nixdorf Recall Study: Identifiability of phone numbers as the major determinant of response. Eur. J. Epidemiol..

[29] Bolke E (2011). Cystatin C—A fast and reliable biomarker for glomerular filtration rate in head and neck cancer patients. Strahlentherapie und Onkologie Organ der Deutschen Rontgengesellschaft ... [et al]..

[30] UNESCO. *International standard classification of education. ISCED 1997.* http://www.unesco.org/education/information/nfsunesco/doc/isced_1997.htm (1997).

[31] Asghar Zaidi M, H. A., de Voss K. *Poverty statistics in the late 1980s: Research base on micro-date.* https://op.europa.eu/en/publication-detail/-/publication/9c787f17-acb6-4f4b-badc-49a2310e65f7 (1995).

[32] Chobanian AV (2003). Seventh report of the Joint National Committee on Prevention, Detection, Evaluation, and Treatment of High Blood Pressure. Hypertension.

[33] Nelson ML, Buchanan-Peart KR, Oribhabor GI, Khokale RV, Cancarevic I (2020). Survival of the fittest: Addressing the disparities in the burden of chronic kidney disease. Cureus.

[34] Peralta CA (2006). African ancestry, socioeconomic status, and kidney function in elderly African Americans: A genetic admixture analysis. J. Am. Soc. Nephrol..

[35] Tamrat R (2015). Apolipoprotein L1, income and early kidney damage. BMC Nephrol..

[36] Thio CHL (2018). Educational level and risk of chronic kidney disease: Longitudinal data from the PREVEND study. Nephrol. Dialysis Transplant..

[37] Canney M (2018). Kidney disease in women is associated with disadvantaged childhood socioeconomic position. Am. J. Nephrol..

