# Supplementary material for: Association between socioeconomic position and cystatin C in the Heinz Nixdorf Recall Study
Source: Sci Rep. 2021 Sep 29;11:19387. doi: 10.1038/s41598-021-98835-7 (PMC8481271; doi:10.1038/s41598-021-98835-7)
Supplement: Supplementary file 1 — Supplementary Tables. [file 41598_2021_98835_MOESM1_ESM.docx]

**SUPPLEMENT**

**Association between Socioeconomic Position and Cystatin C in the Heinz Nixdorf Recall Study**

Tanja Zamrik^1^, Mirjam Frank^1^, Carina Emmel^1^, Lars Christian Rump^2^, Raimund Erbel^1^, Karl-Heinz Jöckel^1^, Nico Dragano^3^, Börge Schmidt^1#^

^1^ Institute for Medical Informatics, Biometry and Epidemiology, University of Duisburg-Essen, Germany

^2^ Clinic of Nephrology, University Hospital Düsseldorf, Germany.

^3^ Institute of Medical Sociology, Centre for Health and Society, University Hospital Düsseldorf, Germany

# Corresponding Author:

Börge Schmidt

Institute for Medical Informatics, Biometry and Epidemiology

University Hospital Essen

Hufelandstr. 55

45122 Essen

Germany

e-Mail: boerge.schmidt@uk-essen.de

Table S1: Beta estimates and 95% confidence intervals (95% CI) for the association of education (per 5 years of education) with cystatin C in linear regression models excluding participants with coronary artery disease, stroke or GFR < 60 ml/min/1,73m^2^ (basic model: adjusted for age (+ sex); full model: age (+ sex), BMI, hypertension, diabetes, hs-CRP, total cholesterol, HDL, triglycerides, smoking).

|  | All | Men | Women |
| --- | --- | --- | --- |
|  | \| N \| β \| 95% CI \| \| --- \| --- \| --- \| | \| N \| β \| 95% CI \| \| --- \| --- \| --- \| | \| N \| β \| 95% CI \| \| --- \| --- \| --- \| |
| Crude model | \| 3718 \| -0.014 \| -0.024 - -0.004 \| \| --- \| --- \| --- \| | \| 1835 \| -0.022 \| -0.037 - -0.008 \| \| --- \| --- \| --- \| | \| 1883 \| -0.045 \| -0.059 - -0.032 \| \| --- \| --- \| --- \| |
| Basic model | \| 3718 \| -0.015 \| -0.024 - -0.005 \| \| --- \| --- \| --- \| | \| 1835 \| -0.013 \| -0.027 - 0.000 \| \| --- \| --- \| --- \| | \| 1883 \| -0.016 \| -0.030 - -0.003 \| \| --- \| --- \| --- \| |
| Full model | \| 3686 \| -0.002 \| -0.012 - 0.007 \| \| --- \| --- \| --- \| | \| 1816 \| -0.003 \| -0.017 - 0.011 \|  \| \| --- \| --- \| --- \| --- \| | \| 1870 \| -0.002 \| -0.014 - 0.011 \| \| --- \| --- \| --- \| |

Table S2: Beta estimates and 95% confidence intervals (95% CI) for the association of income (per 1000 Euro/month) with cystatin C in linear regression models excluding participants with coronary artery disease, stroke or GFR < 60 ml/min/1,73m^2^ (basic model: adjusted for age (+ sex); full model: age (+ sex), BMI, hypertension, diabetes, hs-CRP, total cholesterol, HDL, triglycerides, smoking).

|  | All | Men | Women |
| --- | --- | --- | --- |
|  | \| N \| β \| 95% CI \| p-Wert \| \| --- \| --- \| --- \| --- \| | \| N \| β \| 95% CI \| p-Wert \| \| --- \| --- \| --- \| --- \| | \| N \| β \| 95% CI \| p-Wert \| \| --- \| --- \| --- \| --- \| |
| Crude model | \| 3494 \| -0.013 \| -0.020 - -0.006 \| <0,01 \| \| --- \| --- \| --- \| --- \| | \| 1767 \| -0.016 \| -0.025 - -0.007 \| <0,01 \| \| --- \| --- \| --- \| --- \| | \| 1727 \| -0.020 \| -0.030 - -0.011 \| <0,01 \| \| --- \| --- \| --- \| --- \| |
| Basic model | \| 3494 \| -0.010 \| -0.017 - -0.004 \| <0,01 \| \| --- \| --- \| --- \| --- \| | \| 1767 \| -0.010 \| -0.019 - -0.001 \| 0,02 \| \| --- \| --- \| --- \| --- \| | \| 1727 \| -0.011 \| -0.019 - -0.002 \| 0,02 \| \| --- \| --- \| --- \| --- \| |
| Full model | \| 3465 \| -0.005 \| -0.011 - 0.001 \| 0,12 \| \| --- \| --- \| --- \| --- \| | \| 1748 \| -0.006 \| -0.015 - 0.003 \| 0,21 \| \| --- \| --- \| --- \| --- \| | \| 1717 \| -0.003 \| -0.011 - 0.005 \| 0,44 \| \| --- \| --- \| --- \| --- \| |

### Table S3: Beta estimates and 95% confidence intervals (95% CI) for the association of education categories (≥18 years of education as reference) with cystatin C in linear regression models excluding participants with coronary artery disease, stroke or GFR < 60 ml/min/1,73m^2^ (basic model: adjusted for age (+ sex); full model: age (+ sex), BMI, hypertension, diabetes, hs-CRP, total cholesterol, HDL, triglycerides, smoking).

|  |  | $\leq$10 years of education | 11 - 13 years of education | 14 - 17 years of education |
| --- | --- | --- | --- | --- |
|  | N | \| β \| 95% CI \| p-Wert \| \| --- \| --- \| --- \| | \| β \| 95% CI \| p-Wert \| \| --- \| --- \| --- \| | \| β \| 95% CI \| p-Wert \| \| --- \| --- \| --- \| |
| **All** |  |  |  |  |
| Crudes model | 3718 | \| 0.044 \| 0.024 - 0.063 \| <0,01 \| \| --- \| --- \| --- \| | \| 0.010 \| -0.004 - 0.025 \| 0,16 \| \| --- \| --- \| --- \| | \| 0.014 \| -0.002 - 0.031 \| 0,09 \| \| --- \| --- \| --- \| |
| Basic model | 3718 | \| 0.027 \| 0.008 - 0.046 \| <0,01 \| \| --- \| --- \| --- \| | \| 0.004 \| -0.010 - 0.018 \| 0,55 \| \| --- \| --- \| --- \| | \| -0.006 \| -0.022 - 0.009 \| 0,43 \| \| --- \| --- \| --- \| |
| Full model | 3686 | \| 0.002 \| -0.017 - 0.020 \| 0,10 \| \| --- \| --- \| --- \| | \| -0.014 \| -0.028 - -0.001 \| 0,03 \| \| --- \| --- \| --- \| | \| -0.020 \| -0.035 - -0.005 \| <0,01 \| \| --- \| --- \| --- \| |
| **Men** |  |  |  |  |
| Crude model | 1835 | \| 0.048 \| 0.013 - 0.083 \| <0,01 \| \| --- \| --- \| --- \| | \| 0.023 \| 0.003 - 0.043 \| 0,03 \| \| --- \| --- \| --- \| | \| 0.010 \| -0.011 - 0.031 \| 0,35 \| \| --- \| --- \| --- \| |
| Basic model | 1835 | \| 0.029 \| -0.004 - 0.062 \| 0,09 \| \| --- \| --- \| --- \| | \| 0.005 \| -0.014 - 0.024 \| 0,59 \| \| --- \| --- \| --- \| | \| -0.006 \| -0.026 - 0.014 \| 0,58 \| \| --- \| --- \| --- \| |
| Full model | 1816 | \| 0.009 \| -0.024 - 0.042 \| 0,70 \| \| --- \| --- \| --- \| | \| -0.012 \| -0.031 - 0.007 \| 0,28 \| \| --- \| --- \| --- \| | \| -0.018 \| -0.037 - 0.002 \| 0,06 \| \| --- \| --- \| --- \| |
| **Women** |  |  |  |  |
| Crude model | 1883 | \| 0.075 \| 0.050 - 0.100 \| <0,01 \| \| --- \| --- \| --- \| | \| 0.024 \| 0.003 - 0.046 \| 0,03 \| \| --- \| --- \| --- \| | \| 0.001 \| -0.026 - 0.028 \| 0,93 \| \| --- \| --- \| --- \| |
| Basic model | 1883 | \| 0.027 \| 0.002 - 0.052 \| 0,04 \| \| --- \| --- \| --- \| | \| 0.003 \| -0.018 - 0.024 \| 0,79 \| \| --- \| --- \| --- \| | \| -0.007 \| -0.033 - 0.018 \| 0,57 \| \| --- \| --- \| --- \| |
| Full model | 1870 | \| -0.002 \| -0.026 - 0.022 \| 0,56 \| \| --- \| --- \| --- \| | \| -0.017 \| -0.038 - 0.003 \| 0,03 \| \| --- \| --- \| --- \| | \| -0.022 \| -0.046 - 0.003 \| 0,05 \| \| --- \| --- \| --- \| |

### Table S4: Beta estimates and 95% confidence intervals (95% CI) for the association of income quartiles (4^th^ quartile as reference) with cystatin C in linear regression models excluding participants with coronary artery disease, stroke or GFR < 60 ml/min/1,73m^2^ (basic model: adjusted for age (+ sex); full model: age (+ sex), BMI, hypertension, diabetes, hs-CRP, total cholesterol, HDL, triglycerides, smoking).

|  |  | 1^st^ quartile | 2^nd^ quartile | 3^rd^ quartile |
| --- | --- | --- | --- | --- |
|  | N | \| β \| 95% CI \| p-Wert \| \| --- \| --- \| --- \| | \| β \| 95% CI \| p-Wert \| \| --- \| --- \| --- \| | \| β \| 95% CI \| p-Wert \| \| --- \| --- \| --- \| |
| **All** |  |  |  |  |
| Crude model | 3494 | \| 0.026 \| 0.013 - 0.038 \| <0,01 \| \| --- \| --- \| --- \| | \| 0.022 \| 0.007 - 0.037 \| <0,01 \| \| --- \| --- \| --- \| | \| 0.019 \| 0.006 - 0.032 \| <0,01 \| \| --- \| --- \| --- \| |
| Basic model | 3494 | \| 0.016 \| 0.004 - 0.028 \| <0,01 \| \| --- \| --- \| --- \| | \| 0.019 \| 0.005 - 0.033 \| <0,01 \| \| --- \| --- \| --- \| | \| 0.011 \| -0.001 - 0.023 \| 0,07 \| \| --- \| --- \| --- \| |
| Full model | 3465 | \| 0.005 \| -0.006 - 0.017 \| 0,38 \| \| --- \| --- \| --- \| | \| 0.012 \| -0.002 - 0.025 \| 0,11 \| \| --- \| --- \| --- \| | \| 0.004 \| -0.008 - 0.016 \| 0,54 \| \| --- \| --- \| --- \| |
| **Men** |  |  |  |  |
| Crude model | 1767 | \| 0.035 \| 0.017 - 0.054 \| <0,01 \| \| --- \| --- \| --- \| | \| 0.019 \| -0.003 - 0.040 \| 0,10 \| \| --- \| --- \| --- \| | \| 0.013 \| -0.005 - 0.030 \| 0,16 \| \| --- \| --- \| --- \| |
| Basic model | 1767 | \| 0.019 \| 0.001 - 0.036 \| 0,04 \| \| --- \| --- \| --- \| | \| 0.011 \| -0.009 - 0.032 \| 0,28 \| \| --- \| --- \| --- \| | \| 0.001 \| -0.016 - 0.017 \| 0,93 \| \| --- \| --- \| --- \| |
| Full model | 1748 | \| 0.010 \| -0.008 - 0.027 \| 0,30 \| \| --- \| --- \| --- \| | \| 0.008 \| -0.012 - 0.028 \| 0,52 \| \| --- \| --- \| --- \| | \| -0.004 \| -0.020 - 0.013 \| 0,60 \| \| --- \| --- \| --- \| |
| **Women** |  |  |  |  |
| Crude model | 1727 | \| 0.037 \| 0.019 - 0.054 \| <0,01 \| \| --- \| --- \| --- \| | \| 0.043 \| 0.023 - 0.063 \| <0,01 \| \| --- \| --- \| --- \| | \| 0.032 \| 0.013 - 0.050 \| <0,01 \| \| --- \| --- \| --- \| |
| Basic model | 1727 | \| 0.017 \| 0.001 - 0.034 \| 0,04 \| \| --- \| --- \| --- \| | \| 0.027 \| 0.008 - 0.046 \| <0,01 \| \| --- \| --- \| --- \| | \| 0.025 \| 0.008 - 0.043 \| <0,01 \| \| --- \| --- \| --- \| |
| Full model | 1717 | \| 0.002 \| -0.014 - 0.018 \| 0,74 \| \| --- \| --- \| --- \| | \| 0.016 \| -0.002 - 0.034 \| 0,11 \| \| --- \| --- \| --- \| | \| 0.012 \| -0.005 - 0.029 \| 0,14 \| \| --- \| --- \| --- \| |
